# Supplementary material for: Effects of Bile Acids on Growth Performance and Lipid Metabolism during Chronic Heat Stress in Broiler Chickens
Source: Animals (Basel). 2021 Feb 27;11(3):630. doi: 10.3390/ani11030630 (PMC7997420; doi:10.3390/ani11030630)
Supplement: Supplementary file 1 [file animals-11-00630-s001.pdf]

Table S1. Ingredient composition of the experimental diets (As-fed basis)

Items	0 ~ 21d	21 ~ 42d	21 ~ 42d
	Basal diet	Basal diet	BA diet
Ingredients (%)			
Corn	58.38	64.06	64.06
Soybean meal	27.50	22.00	22.00
Corn gluten meal	5.00	5.00	5.00
Soybean oil	4.00	4.00	4.00
$CaHPO_4$	1.80	1.80	1.80
NaCl	0.40	0.30	0.30
Limestone	1.30	1.30	1.30
Choline chloride	0.10	0.10	0.10
L-Lysine-HCl	0.22	0.18	0.18
DL- Methionine	0.20	0.16	0.16
L- Threonine	0.10	0.10	0.10
Premix[1]	1.00	1.00	1.00
Bile Acid Compound	-	-	0.02

[1] The nutrients provided per kilogram of premix as follows. Vitamin A, 5000 IU; Vitamin D, 3000 IU; Vitamin E, 75 mg; Vitamin K_3, 18.8 mg; Vitamin B_1, 9.8 mg; Vitamin B_2, 28.8 mg; Vitamin B_6, 19.6 mg; Vitamin B_{12} , 0.1 mg; Calcium pantothenate, 58.8 mg; Niacin, 196.0 mg; Folic acid, 4.9 mg; Biotin, 2.5 mg; Cu (as copper sulfate), 4.0 mg; Fe (as ferrous sulfate), 40.0 mg; Zn (as zinc sulfate), 37.6 mg; Mn (as manganese sulfate), 50.0 mg; Se (as sodium selenite), 0.2 mg; I (as potassium iodide), 0.2 mg.

Table S2. Primers of the lipid metabolism and bile acid synthesis related genes in liver

Gene[1]	GenBank ID	Primer sequence
FXR	NM_204113.2	F:5′-CAGAAAGAATGCAGCGGCTC-3′
		R:5′-CAAACTGCCCCATTTTGCGA-3′
FAS	NM_205155.3	F:5′-GCTAAGATGGCATTGCACGG-3′
		R:5′-TGCCAGAGCCTCCACTATCT-3′
SREBP-1c	XM_015294109.2	F:5′-AGGCGGAGGTGATGGAGAT-3′
		R:5′-TCGGAGTCACTGCTGCTGTT-3′
ApoB	NM_001044633.1	F: 5′-GGTTACTCCCACGATGGCAA-3′
		R:5′-TCGCAGAAATGCCCTTCCTT-3′
HMGCR	NM_204485.2	F: 5′-CTCGGCCGGGCGATTTG-3′
		R:5′-AAGTTGTCGCACTCCTGACAT-3′
CYP7A1	NM_001001753.1	F: 5′-TGCTCCGCATGTTCCTGAAT-3′
		R:5′-AGAAGGTAAACAAGCTCCAAAAAGT-3′
CYP8B1	XM_025147312.1	F: 5′-CCTTTCGGAGACGAAGACCC-3′
		R:5′-AAGTCCAGTGCGTAACCCAG-3′
CYP27A1	XM_422056.6	F: 5′-ACTTTCGTCTGGCTCTTCCTG-3′
		R:5′-GTGGCCAAAGGTTGACTTCC-3′
GAPDH	NM_204305.1	F: 5′-GGCACTGTCAAGGCTGAGAA-3′
		R:5′-CACCTGCATCTGCCCATTTG-3′

[1]SREBF1, sterol regulatory element binding transcription factor 1; FAS, fatty acid synthase; SREBP-1c,sterol regulatory element binding protein-1c; ApoB, apolipoprotein B; HMGCR,3-hydroxy-3-methylglutaryl-coenzyme A reductase; CYP7A1, cholesterol 7α-hydroxylyase; CYP8B1, sterol 12α-hydroxylase; CYP27A1, sterol 27-hydroxylase; GAPDH was used as an internal control for normalizing the mRNA levels of tested gene.

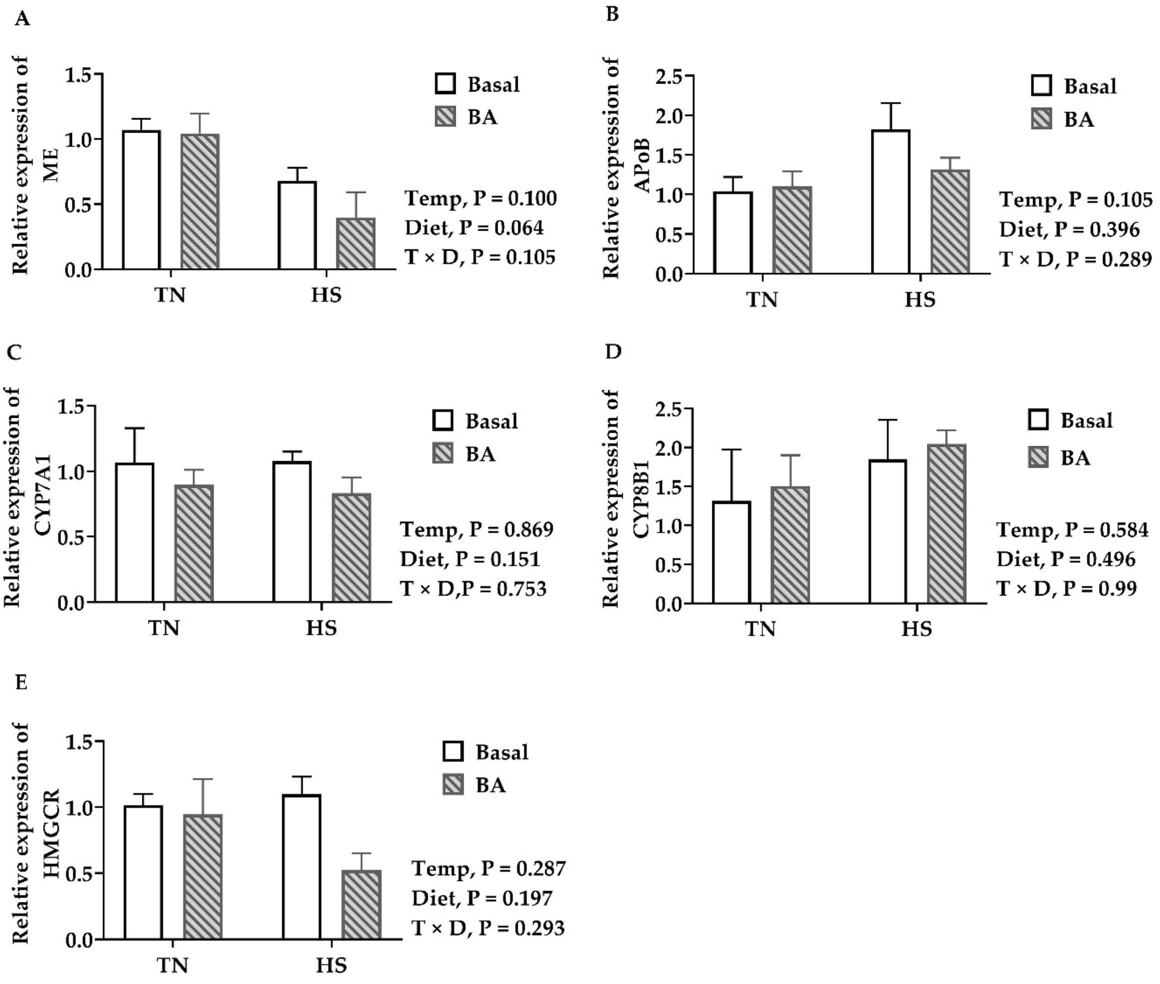

Figure 1. Gene expressions in the liver of broilers fed with 0 or 200 mg/kg bile acids under thermoneutral (TN) and heat-stressed (HS) conditions. (A) ME, melic enzyme; (B) ApoB, apolipoprotein B; (C) CYP7A1, cholesterol 7α-hydroxylase; (D) CYP8B1, sterol 12α-hydroxylase ;(E) HMGCR, 3-hydroxy-3-methyl glutaryl coenzyme A reductase. Data were the means ± SEM (n = 6 for each group). $^{*}P < 0.05$.
